# *Helicobacter pylori* Primary and Secondary Genotypic Resistance to Clarithromycin and Levofloxacin Detection in Stools: A 4-Year Scenario in Southern Italy

**DOI:** 10.3390/antibiotics9100723

**Published:** 2020-10-21

**Authors:** Giuseppe Losurdo, Floriana Giorgio, Maria Pricci, Bruna Girardi, Francesco Russo, Giuseppe Riezzo, Manuela Martulli, Mariano Piazzolla, Francesco Cocomazzi, Francesco Abbruzzi, Elisabetta Parente, Rosa Paolillo, Alessia Mileti, Andrea Iannone, Mariabeatrice Principi, Enzo Ierardi, Alfredo Di Leo

**Affiliations:** 1 Section of Gastroenterology, Department of Emergency and Organ Transplantation, University “Aldo Moro” of Bari, 70124 Bari, Italy; giuseppelos@alice.it (G.L.); mariano.piazzolla@gmail.com (M.P.); francescococomazzi@gmail.com (F.C.); fabbru89@gmail.com (F.A.); eliparente90@gmail.com (E.P.); rosina.paolillo@gmail.com (R.P.); alessia.mileti@hotmail.it (A.M.); ianan@hotmail.it (A.I.); b.principi@gmail.com (M.P.); ierardi.enzo@gmail.com (E.I.); 2 Ph.D. Course in Organs and Tissues Transplantation and Cellular Therapies, Department of Emergency and Organ Transplantation, University “Aldo Moro” of Bari, 70124 Bari, Italy; 3 THD SpA, 42015 Correggio, Italy; flomic@libero.it (F.G.); mirellapricci@libero.it (M.P.); brunagirardi@virgilio.it (B.G.); 4 Laboratory of Nutritional Pathophysiology, National Institute of Gastroenterology “S. de Bellis” Research Hospital, 70013 Castellana Grotte, Italy; francesco.russo@irccsdebellis.it (F.R.); giuseppe.riezzo@irccsdebellis.it (G.R.); manuela.martulli@irccsdebellis.it (M.M.)

**Keywords:** *Helicobacter pylori*, clarithromycin, levofloxacin, antibiotic resistance, molecular analysis, stools

## Abstract

Antibiotic resistance has become an emerging problem for treating *Helicobacter pylori* (*H. pylori*) infection. Clarithromycin and levofloxacin are two key antibiotics used for its eradication. Therefore, we reviewed our experience with genotypic resistance analysis in stools to both clarithromycin and levofloxacin in the last four years to evaluate time trends, both in naive and failure patients. Patients collected a fecal sample using the THD fecal test device. Real-time polymerase chain reaction was performed to detect point mutations conferring resistance to clarithromycin (A2142C, A2142G, and A2143G in *23S rRNA*) and levofloxacin (substitutions at amino acid position 87 and 91 of *gyrA*). One hundred and thirty-five naive patients were recruited between 2017–2020. Clarithromycin resistance was detected in 37 (27.4%). The time trend did not show any significant variation from 2017 to 2020 (*p* = 0.33). Primary levofloxacin resistance was found in 26 subjects (19.2%), and we observed a dramatic increase in rates from 2017 (10%) to 2018 (3.3%), 2019 (20%), and 2020 (37.8%). Ninety-one patients with at least one eradication failure were recruited. Secondary resistance to clarithromycin and levofloxacin was found in 59 (64.8%) and 45 patients (59.3%), respectively. In conclusion, our geographic area has a high risk of resistance to clarithromycin. There is also a progressive spreading of levofloxacin-resistant strains.

## 1. Introduction

In recent years, antibiotic resistance has become an emerging problem for the treatment of *Helicobacter pylori* (*H. pylori*) infection [1,2,3]. Clarithromycin is a macrolide antibiotic which inhibits bacterial protein biosynthesis by binding to bacterial ribosomes. It is commonly used in first line eradication regimens in combination with amoxicillin and a nitroimidazole. Levofloxacin is a fluoroquinolone antibiotic whose mechanism of action is interference with DNA replication. It is commonly used, in combination with amoxicillin, in second line after an eradication failure. Clarithromycin resistance is the main cause of triple therapy failure. Therefore, in areas with resistance rates above 15%, guidelines suggest concomitant or bismuth-containing quadruple therapy [4]. Resistance to clarithromycin is due to a mutation in domain V of the *H. pylori* 23S rRNA region, and three-point mutations, namely A2143G, A2142G, and A2142C, are responsible for 90% of cases of primary clarithromycin resistance in *H. pylori* strains [5,6] in Western countries, albeit the A2143G mutation more deeply reduces the chance of eradication [7,8,9].

Levofloxacin is a fluoroquinolone antibiotic that is usually administered in the second line regimens. In the past, it has even been proposed in the first line in areas with high clarithromycin resistance, where it demonstrated its effectiveness in more than 90% of cases [10]. However, since the use of fluoroquinolones has spread for urinary and respiratory infection, and considering that cross-resistance among fluoroquinolones may occur, a rapid rise in resistance to levofloxacin has taken place, with rates close to 40% in some reports [11]. This rise may have a relevant impact on eradication rates: a meta-analysis showed that successful eradication was achieved in 91.5% of sensitive strains and only 75% of resistant strains [12]. For levofloxacin, mutations in the gyrase A (*gyrA*) gene are responsible for antibiotic resistance [6].

Conventionally, the detection of antimicrobial resistance requires gastric endoscopic sampling with successive culture and E-test. This method has several limitations. First, it is invasive since it requires endoscopy. Second, even in expert hands, culturing *H. pylori* may be challenging, and it may not be technically feasible [13]. Finally, culture cannot detect heteroresistance, differently from the molecular analysis [14,15]. For such reasons real-time polymerase chain reaction (RT-PCR) is becoming more and more popular to assess point mutations conferring antibiotic resistance [16].

Moreover, different groups have already set up systems to detect point mutations in stools. In this setting, the THD fecal test has shown sensitivity and specificity above 90% [17] and a 100% concordance for the point mutations conferring resistance to clarithromycin between stools and gastric biopsy samples [18]. While a recent meta-analysis [19] identified the bacterial 23S ribosomal RNA subunit gene as the most accurate marker for diagnosis of infection using molecular tests on stool samples, with 82% sensitivity and 99% specificity, there are no data about the detection of *gyrA* mutations in stools. On this basis, we retrospectively reviewed our experience about genotypic resistance analysis in stools to both clarithromycin and levofloxacin in the last four years in order to evaluate time trends, both in naive and failure patients.

## 2. Results

### 2.1. Naive Patients

**Figure 1:**
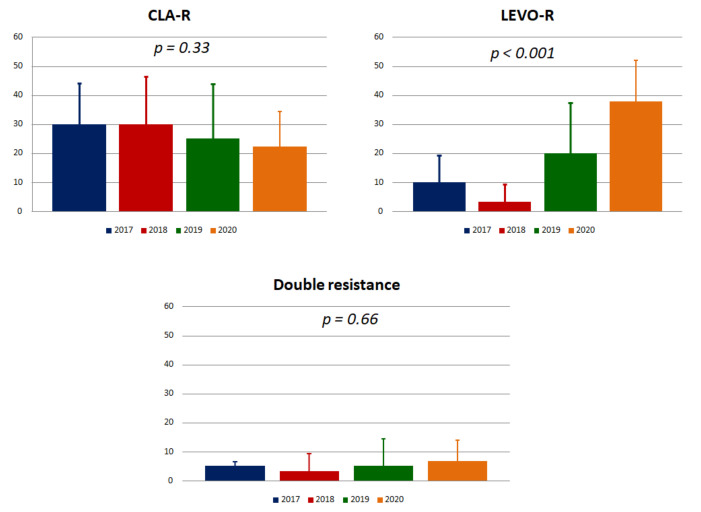
Trends of clarithromycin and levofloxacin resistance in naive patients from 2017 to 2020. Histograms show rates and standard deviation. CLA-R: resistant to clarithromycin; LEVO-R: resistant to levofloxacin.

One hundred thirty-five patients at their first diagnosis of *H. pylori* infection were recruited between 2017 and 2020. Naive patients had not received any therapy for *H. pylori* before. The main demographic and pathologic features are represented in Table 1. Overall, clarithromycin resistance was detected in 37 of them (27.4 ± 7.5%), and all patients had the A2143G mutation. The time trend, reported in Figure 1, did not show any significant variation from 2017 to 2020, with resistance rates ranging from 30% in 2017 to 22.2% in 2020 (*p* = 0.33).

During the three years, levofloxacin resistance was found in 26 subjects (19.2 ± 6.6%). In this case, we observed a dramatic increase in rates from 2017 (10 ± 9.3%) to 2018 (3.3 ± 6.1%), 2019 (20 ± 17.5%), and 2020 (37.8 ± 14.2%), with a *p* < 0.001 (Figure 1).

Double resistance to clarithromycin and levofloxacin was detected in 7 patients (5.2 ± 3.7%), remaining stable below 10% (*p* = 0.66, Figure 1).

### 2.2. Secondary Resistance

Ninety-one patients with at least one eradication failure were recruited. Their relevant demographic and pathologic features are summarized in Table 1. Fifty-six of them had a single failure to clarithromycin-containing regimen (either triple, sequential, or concomitant), while 35 experienced a double failure to both a clarithromycin-containing regimen and levofloxacin-based triple therapy.

**Figure 2:**
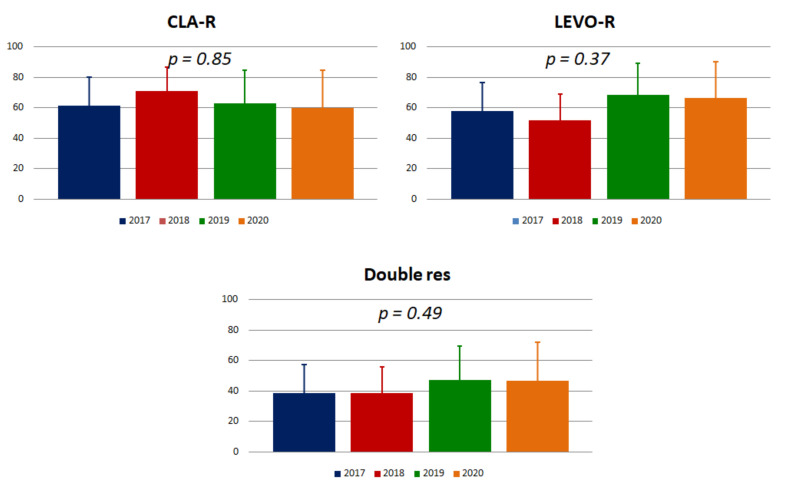
Trends of clarithromycin and levofloxacin resistance in patients with at least one eradication failure from 2017 to 2020. Histograms show rates and standard deviation. CLA-R: resistant to clarithromycin; LEVO-R: resistant to levofloxacin.

Resistance to clarithromycin was found in 59 patients (64.8 ± 9.8%), with the A2143G mutation in all cases. The time trend, reported in Figure 2, was stable from 2017 to 2020 (*p* = 0.85), with resistance constantly above 60%.

Fifty-four patients had resistance to levofloxacin (59.3 ± 10.1%). The highest rate was observed in 2019 (68.4 ± 20.9%) and the lowest in 2018 (51.6 ± 17.6%), without a significant trend (*p* = 0.37, Figure 2).

Double resistance was detected in 38 patients (41.7 ± 10.1%) and constantly remained below 50%, without any time trend across four years (*p* = 0.49, Figure 2).

A comparison between patients with a single failure versus those with two failures showed that resistance to clarithromycin was similar (60.7 versus 71.4%, *p* = 0.37), while patients with two failures had a higher rate of resistance to levofloxacin (88.6% versus 41.1%, *p* < 0.001) and double resistance (57.1% versus 32.1%, *p* = 0.03) than those with one failure (Table 2).

## 3. Discussion

The diffusion of antibiotic resistance has changed the clinical approach of *H. pylori* eradication regimens [2]. The spreading of clarithromycin resistance has led to guideline modifications, suggesting the avoidance of triple therapy in high resistance areas [4]. Therefore, it is fundamental to have up-to-date knowledge about antibiotic resistance rates in each geographic region since this pre-requisite may guide the optimal choice of the most appropriate antibiotics [2,20]. Herein, we showed that in the last four years, clarithromycin resistance was always more than 15%, and this finding confirms previous reports about this antibiotic [21]. While the situation for clarithromycin remained stable, we noticed an impressive rise in levofloxacin resistance in naive patients (Figure 1). This result may be explained by the extensive use of fluoroquinolone agents for other diseases, which could have induced cross resistances. This finding could be a relevant problem since it could limit the use of levofloxacin-based therapies.

Another interesting finding is that patients with a double failure (Table 2) had a high resistance to levofloxacin and double resistance. However, this is an expected result since they have been all treated with levofloxacin-based triple therapy (in contrast to the single failure group).

The present report has several strong points. First, it confirms that genotypic analysis of resistance may be applied as a comprehensive approach, even in a first-line setting. In addition, it may represent a pioneering large-scale attempt to apply noninvasive detection of antibiotic resistance in stools, which may become a relevant procedure in clinical practice. The feasibility and the agreement with standard methods for antibiotic resistance detection have already been proven in previous studies [17,18]. In addition, to the best of our knowledge, this is the first report investigating *gyrA* mutation for levofloxacin resistance to *H. pylori* in stools.

Some limitations should be pointed out. A period of four years could be considered too short for investigating a trend. However, given the recent preparation of the method to extract *H. pylori* DNA from feces and consequent resistance analysis, it includes all patients from inception to July 2020. However, the register will be periodically updated to expand the sample size and draw more consistent results. Furthermore, the study did not explore whether an a priori knowledge of antibiotic resistance could have effectively driven a personalized treatment to increase the eradication rate. In this regard, a trial is ongoing in our center. Finally, we did not have sufficient data about the previous exposure to fluoroquinolone or macrolide antibiotics in patients’ clinical history, and this could be an additional limitation. This is a pivotal point, because previous antibiotic consumption may explain the high prevalence of antibiotic resistance. In this regard, inappropriate antibiotics prescription is the most relevant issue, since a high rate of improper prescription is the main cause for the rise of drug resistance: inappropriate prescription has been estimated to be 64.2% for fluoroquinolones and 74.6% for macrolides [22]. Indeed, a meta-analysis has shown that previous use of any antibiotic is independently associated with resistance, with an odds ratio of 2.3, and this phenomenon was more evident in Southern Europe [23].

In conclusion, our report confirms that our geographic area, Puglia, Southern Italy, has a high risk of resistance to clarithromycin. However, the rise in levofloxacin resistance, even in naive patients, should prompt some consideration about using this antibiotic. Indeed, its extensive utilization has already been stigmatized in Italy [24], and for this reason, levofloxacin should be carefully weighed up for *H. pylori* treatment in consideration of the progressive spreading of resistant strains.

## 4. Materials and Methods

### 4.1. Patient Recruitment

Consecutive patients with *H. pylori* infection, diagnosed by the concordance of at least two tests (urea breath test, stool antigen test, endoscopy with histology, and/or rapid urease test) were recruited. Patients aged > 50 and with alarm signs (weight loss, anemia, hematemesis) underwent upper endoscopy, as suggested by guidelines [25]. Both naive patients and those with at least one therapy failure were included in the study. In detail, we enrolled subjects aged >18 and able to express willingness to participate, presenting with dyspeptic symptoms, such as postprandial fullness, early satiation, epigastric pain, and epigastric burning. We excluded patients with gastric or extra-gastric cancer and those who were unable to express informed consent or refused to participate. Additional exclusion criteria were: therapy with proton pump inhibitors or histamine receptor antagonists within two weeks from enrollment and use of antibiotics or bismuth salts in the previous four weeks. Furthermore, recent chronic diarrhea was another reason for exclusion because it could restrict the proper collection of fecal samples. We did not rule out patients with dysbiosis (except for recent diarrhea due to problems in stool collection) or drugs influencing gut microbiota, since they are not expected to impact on *H. pylori* detection or resistance profiling by RT-PCR.

Patients were recruited from January 2017 to July 2020, starting from when we set up the method to detect both clarithromycin and levofloxacin resistance in stools. Two centers (Gastroenterology Unit, University of Bari, and the National Institute of Gastroenterology “S. de Bellis” Research Hospital) participated in the study.

The study was conducted in agreement with the indications of the Declaration of Helsinki, and the local Ethics Committee approved the protocol (AOU Consorziale Policlinico di Bari, protocol no. 74413, approved 16th November 2016). All patients signed informed consent.

### 4.2. Antibiotic Resistance Analysis

Patients collected a fecal sample using the THD fecal test device (THD Spa, Correggio, Reggio Emilia, Italy). It contains a solution that eliminates RT-PCR inhibiting substances, such as hemoglobin and its degradation products, polysaccharide complexes, heavy metals, and proteins. Additionally, it removes large molecules, such as fibers. The treated solution may finally be taken from the collector and processed for DNA extraction. DNA was extracted using the phenol-chloroform procedure. After this last step, RT-PCR was performed to detect point mutations conferring *H. pylori* resistance to clarithromycin, as previously described and extensively employed and validated [7,8,9,14]. Primers and probes for *gyrA* and 23S rRNA mutations were specifically designed for *H. pylori* genetic sequences. We investigated the A2142C, A2142G, and A2143G point mutations in the bacterium gene encoding for 23S rRNA subunit. We assessed the point mutations (C261A, C261G, G271A, A272G, G271T, and A270T) conferring resistance to levofloxacin in the A-subunit of DNA gyrase (*gyrA*) gene of *H. pylori* by investigating amino acid substitutions at position 87 (asparagine to lysine) and 91 (aspartic acid to glycine, aspartic acid to asparagine, and aspartic acid to tyrosine) of the encoded amino acidic sequence [26,27]. The RT-PCR technique to detect mutations conferring levofloxacin resistance was performed, according to Glocker and Kist 2004 [28]. The final step was the high-resolution melting curve analysis using the CFX96 Touch™ Real-Time PCR machine (BioRad, Hercules, CA, USA). 

### 4.3. Statistical Analysis

Continuous data were expressed as mean ± standard deviation, and categorical variables as proportions/percentages with 95% confidence intervals (CI) calculation. Fisher’s exact test and Student’s *t*-test were used to compare discrete and continuous variables, respectively. Trends in antibiotic resistance were analyzed by the chi-square test for trends. All tests were two-sided, and statistical significance was set at *p* < 0.05. The statistical analysis was performed using the software SPSS version 23.0 for Windows (IBM Corp., Armonk, NY, USA).

## Figures and Tables

**Table 1:** Demographic and pathologic endoscopic features in the enrolled population.

	Naive Patients (*n* = 135)	Failure Patients (*n* = 91)	*p*
**Age (mean ± SD)**	42.1 ± 12.8	43.2 ± 13.5	0.54
**Sex M/F**	54/81	41/50	0.49
**Smokers, *n* (%)**	32 (23.7%)	21 (23.1%)	1
**Endoscopic picture, *n***	(*n* = 106)	(*n* = 72)	0.11
**Normal**	16 (15.1%)	6 (8.3%)	
**Antral erosions**	36 (34.0%)	21 (29.2%)	
**Mucosal hyperemia**	51 (48.1%)	43 (59.4%)	
**PUD**	3 (2.8%)	2 (2.8%)	

PUD: peptic ulcer disease (duodenal in all cases). SD: standard deviation.

**Table 2:** Comparison of antibiotic resistance between patients with one or two therapy failures.

	One Failure N = 56	Two Failures N = 35	*p*
**CLA-R, *n* (%)**	34 (60.7%)	25 (71.4%)	0.37
**LEVO-R, *n* (%)**	23 (41.1%)	31 (88.6%)	<0.001
**Double resistance, *n* (%)**	18 (32.1%)	20 (57.1%)	0.03

CLA-R: resistant to clarithromycin; LEVO-R: resistant to levofloxacin.
